# Misdiagnosed myocarditis in arrhythmogenic cardiomyopathy induced by a homozygous variant of *DSG2*: a case report

**DOI:** 10.3389/fcvm.2023.1150657

**Published:** 2023-05-23

**Authors:** Xuwei Liu, Yue Zhang, Wenjuan Li, Qian Zhang, Letao Zhou, Yimin Hua, Hongyu Duan, Yifei Li

**Affiliations:** ^1^ Key Laboratory of Birth Defects and Related Diseases of Women and Children of MOE, Department of Pediatrics, West China Second University Hospital, Sichuan University, Chengdu, China; ^2^ Department of Nursing, West China Second University Hospital, Sichuan University, Chengdu, China

**Keywords:** DSG2, ACM, myocarditis, genetic sequencing, case report

## Abstract

**Background:**

Arrhythmogenic cardiomyopathy (ACM) is an inherited cardiomyopathy that is rarely diagnosed in infants or young children. However, some significant homozygous or compound heterozygous variants contribute to more severe clinical manifestations. In addition, inflammation of the myocardium and ventricular arrhythmia might lead to misdiagnosis with myocarditis. Here, we describe an 8-year-old patient who had been misdiagnosed with myocarditis. Timely genetic sequencing helped to identify this case as ACM induced by a homozygous variant of *DSG2*.

**Case presentation:**

The proband of this case was an 8-year-old boy who initially presented with chest pain with an increased level of cardiac Troponin I. In addition, the electrocardiogram revealed multiple premature ventricular beats. Cardiac magnetic resonance revealed myocardial edema in the lateral ventricular wall and apex, indicating localized injuries of the myocardium. The patient was primarily suspected to have acute coronary syndrome or viral myocarditis. Whole-exome sequencing confirmed that the proband had a homozygous variation, c.1592T > G, of the *DSG2* gene. This mutation site was regulated by DNA modification, which induced amino acid sequence changes, protein structure effects, and splice site changes. According to MutationTaster and PolyPhen-2 analyses, the variant was considered a disease-causing mutation. Next, we used SWISS-MODEL to illustrate the mutation site of p.F531C. The ensemble variance of p.F531C indicated the free energy changes after the amino acid change.

**Conclusion:**

In summary, we reported a rare pediatric case initially presenting as myocarditis that transitioned into ACM during follow-up. A homozygous genetic variant of DSG2 was inherited in the proband. This study expanded the clinical feature spectrum of DSG2-associated ACM at an early age. Additionally, the presentation of this case emphasized the difference between homozygous and heterozygous variants of desmosomal genes in disease progression. Genetic sequencing screening could be helpful in distinguishing unexplained myocarditis in children.

## 1. Introduction

Arrhythmogenic right ventricular cardiomyopathy (ARVC, OMIM:#610476) is an inherited heart muscle disease characterized by the loss of the ventricular myocardium and fibrofatty replacement, which predisposes patients to fatal ventricular arrhythmias and sudden cardiac death (SCD) (1, 2). A genetic etiology has been identified for most inherited cardiovascular diseases, especially cardiomyopathies, with the rapid development of sequencing techniques. Regarding the molecular mechanism, ARVC has been identified as being related to pathogenetic variants in desmosomes and adherens junctions, which are critical for establishing cell‒cell junctions and maintaining intercellular communication (3, 4). Thus, the disease group ACM should be considered to define the broader spectrum of the phenotypic expressions of the disease (4). From a molecular perspective, multiple genes encoding desmosomal proteins, such as plakophilin-2 (*PKP2*), desmoplakin (*DSP*)*, DSG2*, desmocollin (*DSC2*), and plakoglobin (*JUP*), account for 50% of patients with ACM in different cohorts (5, 6). However, there are other genetic (non-desmosomal) and non-genetic causes of the disease. The non-desmosomal genes include *DES*, *LMNA*, *SCN5A*, *PLN*, *TMEM43*, and *TGFB3*, which are not involved in the molecular formation of desmosomes and participate in several types of ARVC origins (2). The inclusion of sarcomere-, ion transporter-, and cytokine-related genes would increase the percentage of patients positive for molecular characterization. However, the incomplete dominance and variable expressiveness of certain variants suggest that environmental factors play an important role. Initially, ARVC was considered to mainly cause right ventricular lesions and impair the function of the right ventricle. However, with the development of sequencing analysis and clinical imaging screening techniques, such as cardiac magnetic resonance (CMR), it has been found that biventricular or even left ventricular dysfunction is the dominant phenotype in ARVC, especially for *DSP* and *DSG2* mutations (2).

Large-sample studies have shown that individuals with more than one mutation may have poorer clinical outcomes, with a fivefold increased risk of developing left ventricular dysfunction and heart failure compared with patients with a single mutation (6). Recently, several types of ACM, especially those involving the left-dominant and biventricular forms, presented atypical and diverse phenotypes. Studies have demonstrated a spectrum of biventricular and left-dominant forms that could be misdiagnosed as myocarditis, unexplained myocardial injuries, or even acute coronary syndrome (ACS). Furthermore, case reports have identified patients who were erroneously diagnosed with myocarditis instead of ARVC, or vice versa, highlighting the clinical and diagnostic overlap (7, 8). There is increasing evidence that underlying genetic abnormalities associated with cardiomyopathy may predispose patients to myocarditis or other myocardial injuries (9). Brodehl et al. (10) demonstrated that genetic mouse models of ACM can exhibit early inflammation between 2 and 3.5 weeks of age. Herein, we report a rare case that initially presented as myocarditis. However, we failed to identify any lesions in his coronary arteries. Molecular genetic analysis revealed a homozygous mutation of *DSG2*, and the proband presented with ACM after a 1-year follow-up. This report expands the spectrum of the clinical presentation of ACM, especially in homozygous *DSG2* variants.

## 2. Case presentation

### 2.1. Clinical presentation and physical examination

This study was approved by the Ethics Committee of the West China Second Hospital of Sichuan University (approval number 2014–034). Informed consent was obtained from the patient's parents before performing whole-exome sequencing and for the inclusion of the patient's clinical and imaging details in subsequent publications.

The proband was an 8-year-old male admitted to our hospital for 5 days due to severe, recurrent, and persistent chest pain. Moreover, the chest pain attacks became more frequent and his pain levels worsened, which he could not tolerate. He described the acute and repeated chest pain as aching and tightness. Additionally, the patient complained of pain spreading from the chest to the shoulders, upper abdomen, and back. In the last 2 days before hospital admission, the patient suffered severe vomiting more than five times per day. When the chest attacks occurred, he experienced palpitations and slightly reduced activity tolerance. However, he denied any syncope and shortness of breath. There was no fever, cough, or indigestion.

The initial physical examination at the emergency department revealed an acute critical illness involving severe diaphoresis, dizziness, and fatigue, with the patient presenting with a pale face and feeling restless. The heart rate was approximately 75 beats per minute, and irregular premature beats were observed. His blood pressure was 91/52 mmHg. At the same time, the breathing rate slightly increased to 25 breaths per minute. This patient had a normal nutrition status, and his response to external stimulation was also normal. No trauma injuries were found on the body surface. The respiratory movements of both lungs were symmetrical; the breath sounds of both lungs were rough, no significant wet rales were heard in the bilateral lungs, and occasional wheezing sounds were heard. The apex of the heartbeat moved to the lower left, and there was a sense of lift in the precordial area. A mildly enlarged heart boundary was identified, and the rhythm was heterogeneous with premature beats. However, the heart sound was dull, and a level I–II systolic murmur was recorded. The abdomen was soft, the liver was 2 cm below the subcostal margin and 1 cm–2 cm below the xiphoid process, the texture was medium, and the spleen was not palpable subcostally. The muscle strength and tension of the four extremities were normal. The pathological signs and meningeal irritation signs were negative.

The patient suffered influenza A infection 1 year before these chest pain attacks, and myocardial injuries had been identified by an increased level of cardiac troponin I (cTnI). The electrocardiogram (ECG) revealed multiple premature ventricular beats. After a series of myocardial protection treatments had been provided, the patient recovered and did not complain of any other cardiac-related symptoms in the year before this illness. Moreover, his parents denied any positive family history of cardiac attacks or cardiovascular, hypertension, and coronary artery diseases. The parents also denied any history of diabetes and obesity among his family members. No inherited disease had been identified in this family, including any cardiomyopathies and metabolic diseases.

### 2.2. Imaging and laboratory examinations

**Figure 1:**
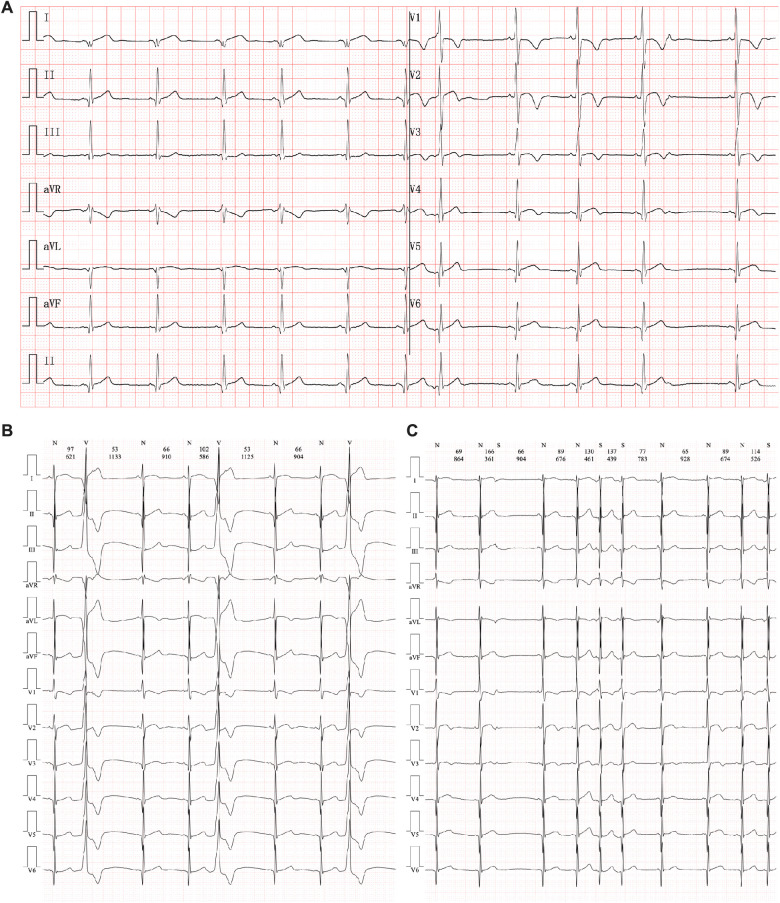
Electrocardiography manifestation in the current proband. (**A**) An abnormal Q wave had been found in II, III, aVF, V5, and V6 leads. (**B**) Multiple premature ventricular beats. (**C**) Paired premature atrial beats.

Routine blood cell tests and blood gas analyses produced results that were within a standard range. In addition, hepatic and renal function tests yielded no significant findings. However, serum cardiac troponin I (2.808 µg/L, n.v. < 0.06 µg/L) and B-type natriuretic peptide (1020.24 pg/ml, n.v. < 100 pg/ml) levels were significantly elevated, demonstrating significant myocardial injury. An ECG was performed immediately, and abnormal Q waves were observed in the II, III, aVF, V5, and V6 leads (Figure 1A). At the same time, Holter scanning revealed multiple ventricular premature beats and paired atrial premature beats. Thus, ACS or viral myocarditis was primarily suspected in this patient. All infection parameters, including CRP, PCT, and ESR, were in the expected range. Additionally, antibody tests of potential viruses involved in myocarditis were assessed, including cytomegalovirus, Epstein–Barr virus, adenovirus, coxsackie virus, and herpes simplex virus, such as human parvovirus B19, and no positive viral infection results were obtained. In addition, autoimmune antibodies and rheumatic tests were performed and all the related results were negative.

**Figure 2:**
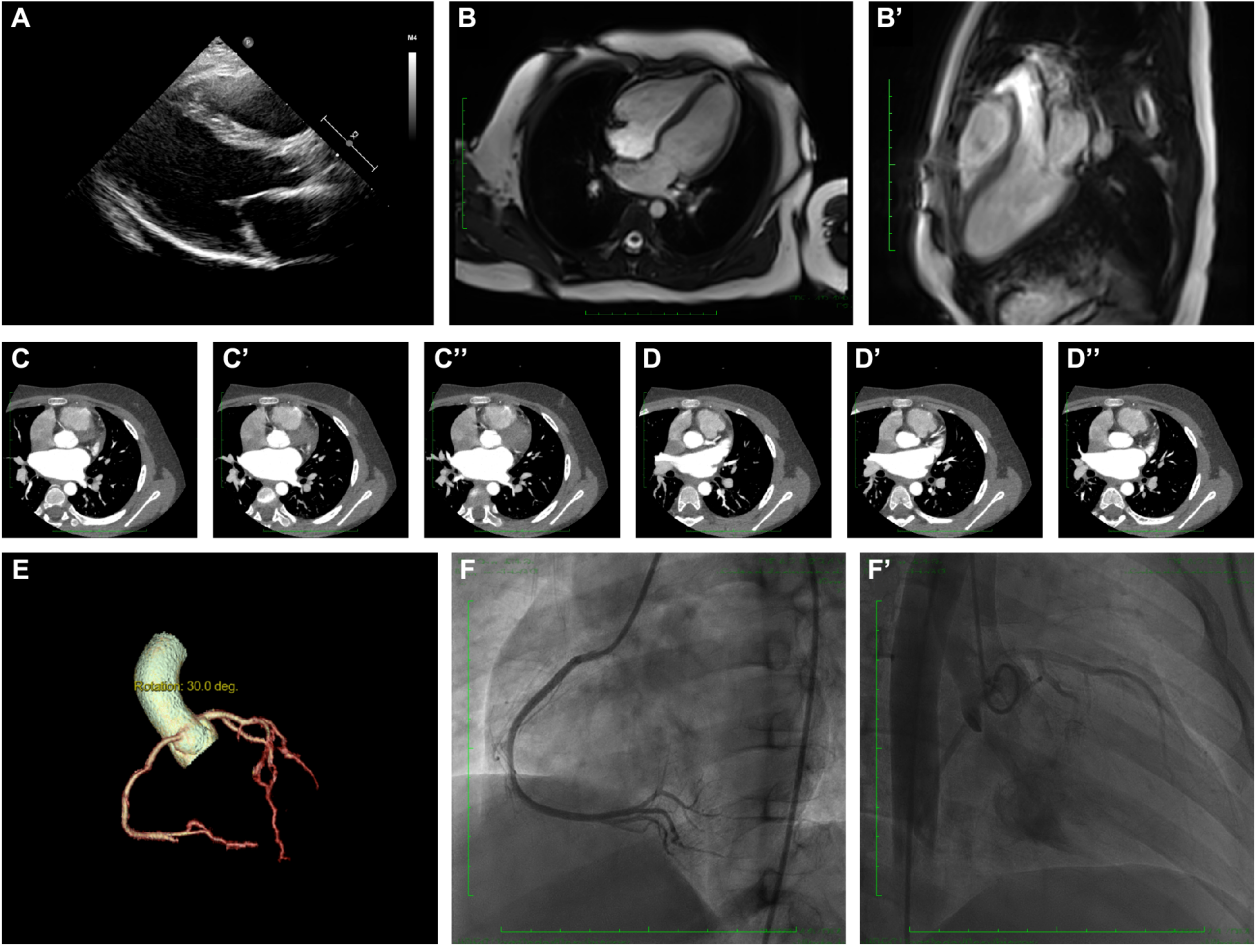
Clinical and radiographic manifestation of the current proband. (**A**) Echocardiography revealed a slight enlargement of the left ventricle. (**B–B’**). Cardiac magnetic resonance showed myocardial edema in the lateral ventricular wall and apex, indicating localized injures of the myocardium. (**C–C”**). CTA revealed normal right coronary artery structure. (**D–D”**) CTA revealed normal left coronary artery structure. (**E**) Coronary artery rebuilding based on CTA. (**F–F’**) Angiographic images of the left coronary artery; no significant positive result was recorded.

Echocardiography demonstrated a slight enlargement of the left ventricle (42 mm), while the left ventricular ejection fraction dropped slightly to 46% at the time he was admitted (Figure 2A). Holter scanning revealed multiple premature ventricular beats and atrial tachycardia, while abnormal Q waves were also identified in the II, III, aVF, and V3-V6 leads (Figures 1B, C). Moreover, repeated ECG examination found an ST-segment elevation among the abovementioned leads, which strongly indicated ACS. Cardiac magnetic resonance (CMR) revealed myocardial edema in the lateral ventricular wall and apex, indicating localized myocardium injuries (Figures 2B, B’). The area of infected myocardium was consistent with the changes in ECG presentation. In addition, CT coronary artery angiography was performed to examine the morphology of the coronary arteries (Figures 2C–E). The right coronary artery was infused and presented a typical structure. Angiographic images of the left coronary artery were also obtained and no significant positive result was recorded (Figures 2F, F’).

Therefore, a myocarditis attack was suspected, while ACS with non-obstructive coronary arteries could not be entirely excluded. Thus, myocardial protection treatment, including creatine phosphate and levocarnitine, and non-invasive mechanical ventilation were provided. Then, antibiotics were administered for potential infection treatment. Dexamethasone was administered to relieve edema of the myocardium. After 2 weeks of treatment and intensive care, the patient recovered from severe chest pain and did not report any discomfort of the heart. After that, the patient was discharged with strict follow-up.

#### 2.3. WES technical method

Owing to the complicated symptoms and negative imaging assessments of this proband, a particular cardiomyopathy was still suspected. Therefore, whole-exome sequencing (WES) was carried out to identify any essential genetic variants. A peripheral blood sample was obtained from the patient in an ethylenediaminetetraacetic acid (EDTA) anticoagulant blood sample tube that was stored at 4°C for less than 6 h. DNA was extracted using a Blood Genome Column Medium Extraction Kit (Tiangen Biotech, Beijing, China). WES was performed using the NovaSeq 6,000 platform (Illumina, San Diego, CA, USA), and the raw data were processed using FastP to remove adapters and filter low-quality reads. Paired-end reads were aligned to the Ensembl GRCh38/hg38 reference genome using the Burrows–Wheeler Aligner. Variant annotation was performed in accordance with database-sourced minor allele frequencies (MAFs) and practical guidelines on pathogenicity issued by the American College of Medical Genetics. The annotation of MAFs was performed according to the 1,000 Genomes, dbSNP, ESP, ExAC, and Chigene inhouse MAF databases and the Provean, Sift, Polypen2_hdiv, and Polypen2_hvar databases using R software (R Foundation for Statistical Computing, Vienna, Austria). To elucidate the molecular architecture of the targeted gene, we used MutationTaster with R software to predict the pathogenicity of the targeted gene and assess the impact of the mutations on protein structure. We performed comparative modeling using SWISS-MODEL. If there was no available full-length protein crystal structure for the targeted gene, the AlphaFold protein structure database (https://alphafold.ebi.ac.uk/) tool was used to predict the protein crystal structure.

### 2.4. Molecular results

**Figure 3:**
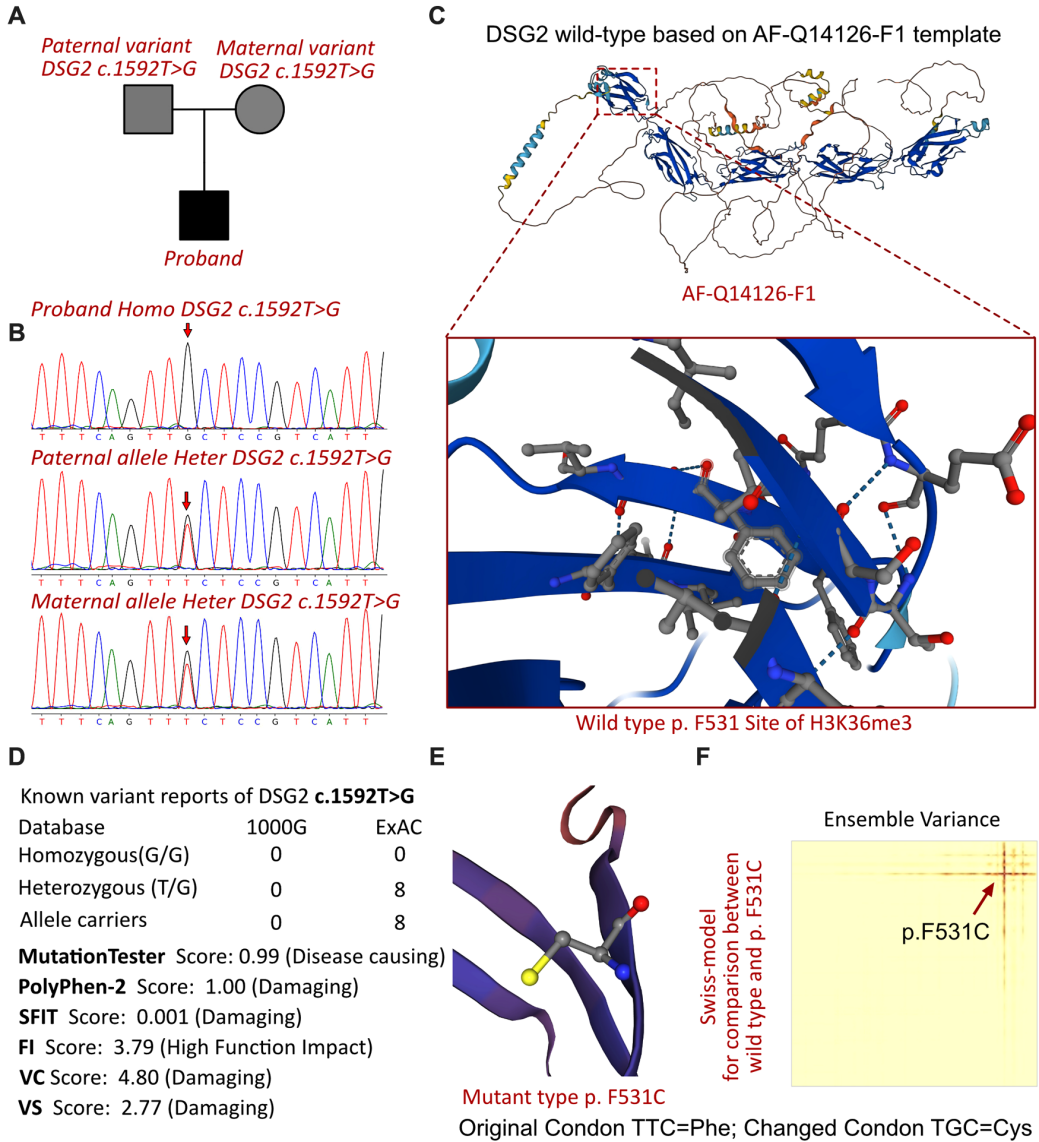
The DSG2 mutations in this family and molecular analysis. (**A**) The proband exhibited a homozygous variant of DSG2 (c.1592T >G; p.F531C). (**B**) Sanger sequencing validation. (**C**) Protein structure predicted by AlphaFold (AF-Q14126-F1). (**D**) The homozygous variant of DSG2 c.1592T >G had never been reported in 1000G and ExAC, while eight records had been retrieved from the ExAC database. Protein damage was predicted by PolyPhen-2 and SFIT. (**E**) Altered amino acid structural site of DSG2 p.F531C. (**F**) Changed free energy on the DSG2 p.F531C site.

A homozygous missense variant in the *DSG2* gene was identified (NM_001943, c.1592T >G; p.F531C). His biological parents were allelic carriers without any clinical manifestations (Figure 3A), and Sanger validation was performed (Figure 3B). Additionally, no other cardiovascular-related variants were retrieved between the proband and his parents. Beyond the reported DSG2 variant, there were no other potential cardiomyopathic variants, and most of them were synonymous mutations. The molecular crystal structure was built in AlphaFold (AF-Q14126-F1, Figure 3C), while the extracellular domain protein structure was used for specific site analysis (5erd.1.A, Figure 3E) (11). In addition, the frequency of this mutation in the population according to database research is presented (Figure 3D), but only a few allele carriers were found in previous reports. This proband would be the youngest identified patient with a homozygous variant change of c.1592T > G in *DSG2*. This mutation site was regulated by DNA modification, which induced amino acid sequence changes, protein structure effects, and splice-site changes. According to MutationTaster analysis, the variant was considered a disease-causing mutation, and the probability value was 0.99. PolyPhen-2 analysis demonstrated a damaging change in this protein (1.00). The SIFT score predicted protein damage (0.001), and MutationAssessor indicated a high molecular function impact on the variant (FI score, 3.79; VC score, 4.80; and VS score 2.77). Then, we used SWISS-MODEL to illustrate the mutation site of p.F531C (Figure 3E) (11). The ensemble variance of p.F531C indicated free energy changes after the amino acid change (Figure 3F).

### 2.5. Final diagnosis, treatment, and follow-Up

During follow-up, the patient presented left ventricular dysfunction (LVEF = 36%) and aggressive enlargement of the left ventricle (48 mm). ECG also identified frequent premature ventricular beats (>1,000 beats per day). Based on the molecular genetic analysis of a homozygous variant of *DSG2*, a diagnosis of arrhythmogenic cardiomyopathy was made, as no significant involvement was found in the right ventricle. After diagnosis, metoprolol and captopril were administered to the patient, who reported no further chest pain and a reduced frequency of arrhythmia. This patient was misdiagnosed as having myocarditis at his initial admission. The results indicate that this homozygous variant of *DSG2* could cause an earlier onset of an adverse cardiac event than heterozygous variants. The first attack of arrhythmogenic cardiomyopathy might result in a series of symptoms, such as ACS. Therefore, genetic screening of unexplained myocarditis is critical.

## 3. Discussion

ACM was once regarded as the most relevant disease in the young adult population. However, an increasing number of pediatric patients, including toddlers and infants, have been identified by advanced imaging and genetic analysis. However, the clinical characteristics and natural history of pediatric ACM are largely unknown. Furthermore, few available data or recommendations have been proposed for administration strategies of ACM in children. We described an 8-year-old child who initially presented with clinical ACS and was subsequently diagnosed with ACM during follow-up due to molecular test results. ACM is considered a rare disease that is probably underestimated due to insufficient awareness of the atypical symptoms, including myocardial injuries, ACS, and myocarditis (12). Left ventricular involvement and biventricular failure were common among homozygous *DSG2* p.F531C variant patients, even at an early age, while heterozygous variant carriers were either unaffected or mild ARVC-related symptoms only presented in 25% of relatives (13). The changes in amino acids were predicted to impair the extracellular domain connections between cardiomyocytes. Patients with ARVC can rarely develop chest pain and ST-segment changes on an ECG, which present similarly to ACS (14). Lopez-Ayala et al. (15) identified 7 out of 195 *DSP* variant carriers who presented with acute myocarditis. The atypical clinical presentation defined as the “hot phase” often occurs in pediatric patients carrying *DSP* and *DSG2* gene mutations (7). Previous studies have suggested that pathogenic *DSP* variants might play a unique role in myocarditis in ACM (16). However, Belkaya et al. (7) demonstrated the enrichment of rare biallelic non-synonymous or splice-site variants in genes associated with inherited cardiomyopathies in a pediatric acute myocarditis cohort (12%) compared with healthy subjects (0.9%). Several specific genetic variants of ACM have been identified in patients with myocarditis-like symptoms. In addition, *DSG2* variants have been recognized as pathogenic variants involved in biventricular impairments, and these variants also contribute to an overlapping manifestation with myocarditis. Moreover, in a recent study, Boogerd et al. (17) found that *PKP2* variants also lead to biventricular dysfunction, which expanded the understanding of ARVC. Brodehl et al. demonstrated two similar cases with *DSG2* and *DSC2* variants (18, 19). This evidence indicated that several extracellular domain impairments of desmosomes would lead to biventricular dysfunction, and it was critical to distinguish them from cardiomyopathies, as they presented a higher risk of SCD. Desmosome-related mutations have been associated with a peculiar phenotype characterized by episodes of acute myocardial injury, induced LV fibrosis, progressive systolic dysfunction, and a high incidence of ventricular arrhythmia (20). These forms are often diagnosed as acute myocarditis. Hata Yukiko et al. (21) revealed that 8 out of 10 cases with unexplained minimal inflammatory foci might be the causative gene variant of cardiomyopathy. Generally, myocarditis has been identified as reduced heart function, changes in ECG, elevated cTnI levels, and abnormal signals in cardiac MRI, according to various guidelines for distinguishing myocarditis. Unfortunately, patients with ACM can also present these clinical manifestations, and several ACM cases were misdiagnosed as myocarditis in practice. Thus, it is urgent to understand the association between different genetic variants in ACM and their overlap with myocarditis.

ACS is seldom observed in pediatric patients. Previous studies demonstrated that familial hypercholesterolemia was the most common cause of pediatric ACS (22). Davlat et al. (23) demonstrated the association of left ventricular non-compaction with ACS. Moreover, Puwanant et al. (24) presented a cohort of 200 patients with hypertrophic cardiomyopathy (HCM) suffering a higher prevalence of ACS. Therefore, it is essential to distinguish the onset of ACS as a significant symptom before the dominant phenotype of cardiomyopathies appears.

Notably, the phenotypic variation between homozygous and heterozygous variant carriers should be addressed. The study of a *DSG2* knockout murine model of ACM revealed cardiac inflammation as a critical early event leading to myocardial fibrosis (25). Modulating inflammatory signaling pathways, such as NF-*κ*B, may be a novel therapeutic target for desmosomal-mediated cardiomyopathy, as recently demonstrated in a mouse model harboring homozygous mutations in *DSG2* (26). Compound/digenic heterozygosity has been identified in up to 25% of patients and has been reported to account for both phenotypic variability and more malignant lifetime arrhythmic outcome (dose effect) (27, 28). While the right-dominant form was typically associated with genes encoding desmosomal proteins, other (non-desmosomal) mutations have been shown to cause biventricular and left-dominant variants. DSG2 mutations have been related to biventricular variants of ACM (29, 30). As several clinical presentations caused by ACM-related genetic variants have recently been identified, the role of genetic mutations in ACM pathogenesis should not be simplified as a linear cause-effect relationship to which a particular phenotype corresponds.

## 4. Conclusion

In summary, we reported a rare pediatric case initially presenting as myocarditis that transitioned into ACM during follow-up. A homozygous genetic variant of *DSG2* was inherited in the proband. This study expanded the clinical feature spectrum of *DSG2*-associated ACM at an early age. Additionally, the presentation of this case emphasized the difference between homozygous and heterozygous variants of desmosomal genes in disease progression. Genetic sequencing screening can be helpful in distinguishing unexplained myocarditis or ACS in children.

## Data Availability

The original contributions presented in the study are included in the article/supplementary materials, further inquiries can be directed to the corresponding authors.
